# Supplementary material for: The TTLL10 polyglycylase is stimulated by tubulin glutamylation and inhibited by polyglycylation
Source: bioRxiv. 2025 Aug 4:2024.03.31.587457. Preprint. [Version 2] doi: 10.1101/2024.03.31.587457 (PMC12340829; doi:10.1101/2024.03.31.587457)
Supplement: Supplement 1 [file NIHPP2024.03.31.587457v2-supplement-1.pdf]

Figure S1. Extracted-ion chromatograms for monoglycylated α1b, βI, and βIVb tubulin tails modified by TTLL8.

(A-C). Extracted-ion chromatograms for monoglycylated α1b-tail peptides with one monoglycine branch (A), two monoglycine branches (B) and three monoglycine branches (C). (B-G). Extracted-ion chromatograms for monoglycylated βI-tail peptides with one monoglycine branch (D), two monoglycine branches (E), three monoglycine branches (F) and four monoglycine branches (G). (H). Extracted-ion chromatograms for monoglycylated βIVb-tail peptides. Tubulin tail peptides were proteolytically released from microtubules incubated with TTLL8.

Figure S2. MS/MS spectra for monoglycylated tubulin C-terminal tail peptides proteolytically excised from microtubules incubated with TTLL8.

(A, B). MS/MS spectra for monoglycylated α1b- peptides (A) and βI-tubulin C-terminal tail peptides (B).

Figure S3. Western blot analysis of glycylation in tubulin purified from tSA201.

bioRxiv preprint doi: https://doi.org/10.1101/2024.03.31.587457; this version posted August 4, 2025. The copyright holder for this preprint (which was not certified by peer review) is the author/funder. This article is a US Government work. It is not subject to copyright under 17 USC 105 and is also made available for use under a CC0 license.

0.2 μgs of purified tubulin were loaded for each unmodified and glycylated sample. Glycylation was detected with the Gly-pep1 and anti-polyG antibodies (Materials and Methods). We note a weak reactivity to the gly-pep1 antibody for the tubulin purified from tSA201 cells. It is unclear whether the antibody detects a very small proportion of mono- or bi-glycylated tubulin in this sample or the signal is due to a low affinity interaction of the antibody for the unmodified tubulin tail which is visible at these tubulin loading levels. Neither our LC-MS or MS/MS data of the tsA201 tubulin detected any glycylation on the intact tubulin, or tubulin tails, respectively. The higher molecular weight band is from TTLL8 which self-modifies during expression. The tSA201 tubulin shows no reactivity against the polyglycylation antibody.

Figure S4. Extracted-ion chromatograms for polyglycylated tubulin C-terminal tail peptides proteolytically excised from monoglycylated microtubules incubated with TTLL10.

(A-C). Extracted-ion chromatograms for polyglycylated α1b- peptides (A), βI- peptides (B) and βIVb-tubulin (C) C-terminal tail peptides. The subscript in G_i indicates the length of the polyglycine chain.

Figure S5. MS/MS spectra for polyglycylated tubulin C-terminal tail peptides proteolytically released from microtubules incubated with TTLL10 show elongation of polyglycine chains only at positions where monoglycine branches were already initiated by TTLL8.

(A, B). MS/MS spectra for α1b- (A) and βI-tubulin (B) C-terminal tail peptides. The subscript in G_i indicates the length of the polyglycine chain.

Figure S6. LC-MS of differentially monoglycylated microtubules used in assays shown in Figure 3.

The weighted mean of the number of glycines ($\langle n^G \rangle$) added to α- and β-tubulin are denoted $\alpha + \langle n^G \rangle$; $\beta + \langle n^G \rangle$. The number of posttranslationally added glycines is indicated in green (for α-tubulin isoforms) and blue (β-tubulin isoforms) on the spectra.

bioRxiv preprint doi: https://doi.org/10.1101/2024.03.31.587457; this version posted August 4, 2025. The copyright holder for this preprint (which was not certified by peer review) is the author/funder. This article is a US Government work. It is not subject to copyright under 17 USC 105 and is also made available for use under a CC0 license.

Figure S7. LC-MS of monoglycylated and polyglycylated microtubules used in binding assays shown in Figure 4.

The weighted mean of the number of glycines ($<n^{G}>$) added to α- and β-tubulin are denoted $\alpha + <n^{G}>$; $\beta + <n^{G}>$.The number of posttranslationally added glycines is indicated in green (for α-tubulin isoforms) and blue (β-tubulin isoforms) on the spectra.

Figure S8. Western blot analysis of glutamylation in tubulin purified from tSA201 cells.

0.2 μg of tubulin was loaded for each unmodified and glutamylated sample. Glutamylation was detected with the GT335 antibody (Materials and Methods). No signal is detectable in the unmodified tsA201 purified tubulin. The MS/MS analysis of this tubulin did not identify any glutamylated peptides, either. In contrast, the tSA201 tubulin enzymatically glutamylated *in vitro* shows strong signal that increases with glutamylation level. Number of posttranslationally added glutamates indicated and determined from LC-MS measurements. The glutamylated tubulin runs slightly higher due to the changes in electrophoretic mobility.

Figure S9. Estimation of glutamylation levels of monoglycylated and polyglycylated microtubules used in microtubule binding assays shown in Figure 5.

(A) Western blot of polyglutamylated and monoglycylated or polyglycylated microtubules used in microtubule binding assays shown in Figure 5. From the left, first four lanes, TTLL6 polyglutamated microtubules with listed mean glutamate numbers determined from LC-MS spectra of intact microtubules. These were used to calibrate the polyglutamate signal detected with an anti-poly-E antibody (clone IN105, Adipogen; Materials and Methods); Subsequent four lanes, dually modified microtubules (glycylated with TTLL8 or TTLL8+10, and glutamylated with TTLL6) used in the assays shown in Figure 5; (B) Poly-E signal as a function of total glutamate numbers on α- and β-tubulin. The signal for the two dually modified species (monoglcylated + polyglutamylated and polyglycylated + polyglutamylated) used in the TIRF-based assays is shown with a discontinuous line.

Figure S1

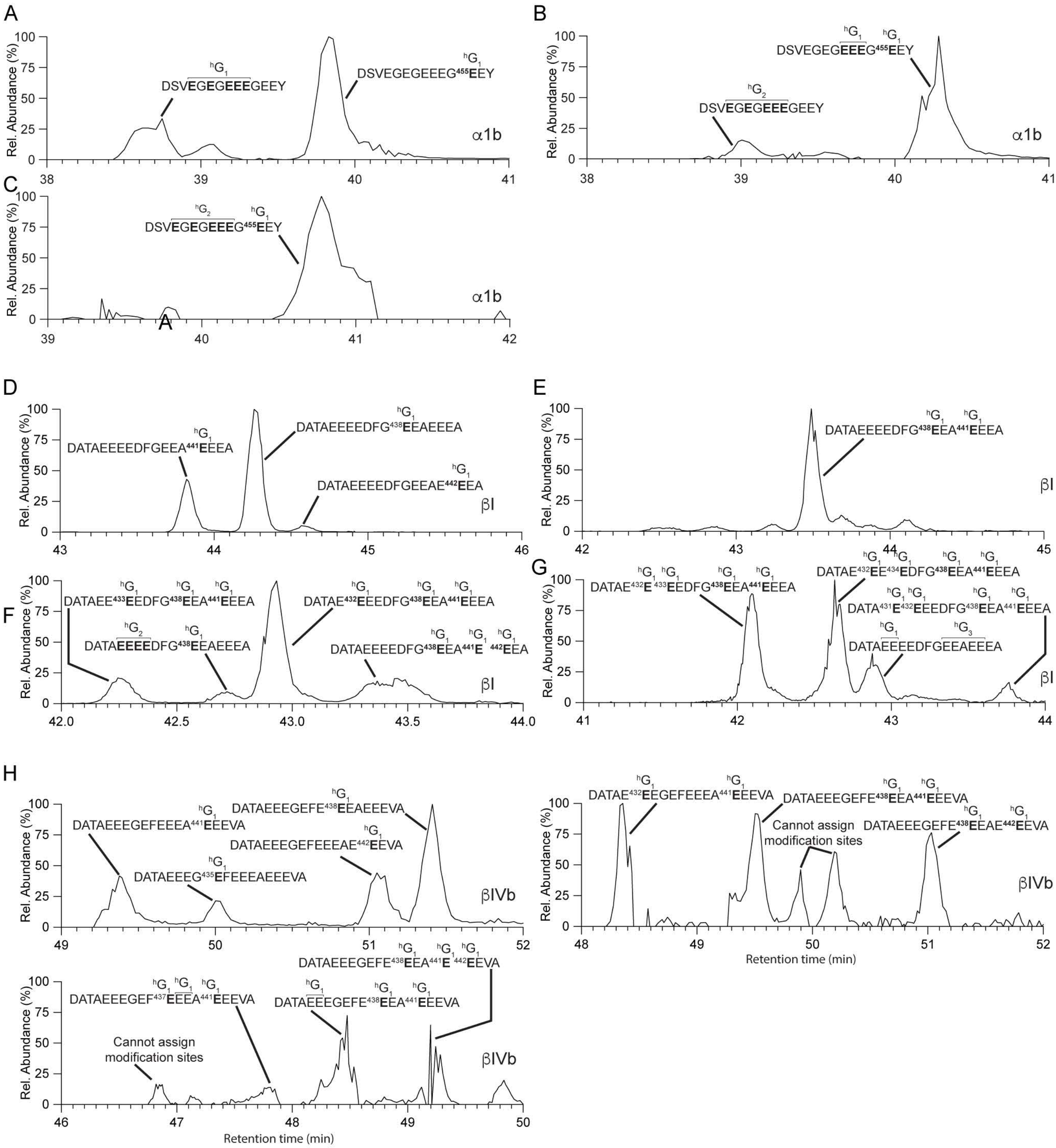
A
B
C
D
E
F
G
H
Rel. Abundance (%)
Retention time (min)
α1b
βI
βIVb
DSVEGEGEEEGEEY
DSVEGEGEEEG455EEY
DSVEGEGEEEGEEY
DSVEGEGEEEG455EEY
DSVEGEGEEEG455EEY
DATAEEEEDFGEEA441EEEA
DATAEEEEDFG438EEAEEEA
DATAEEEEDFGEEAE442EEA
DATAEEEEDFG438EEA441EEEA
DATAEE433EEDFG438EEA441EEEA
DATAEEEEDFG438EEAEEEA
DATAE432EEEDFG438EEA441EEEA
DATAEEEEDFG438EEA441E 442EEA
DATAE432E 433EEDFG438EEA441EEEA
DATAE432EE434EDFG438EEA441EEEA
DATA431E432EEEDFG438EEA441EEEA
DATAEEEEDFGEEEAEEEA
DATAEEEGEFEEEA441EEEVA
DATAEEEGEFE438EEAEEEVA
DATAEEEGEFEEEAE442EEVA
DATAEEEG435EFEEEAEEEVA
DATAE432EEGEFEEEA441EEEVA
DATAEEEGEFE438EEA441EEEVA
Cannot assign modification sites
DATAEEEGEFE438EEAE442EEVA
DATAEEEGEFE438EEA441E442EEVA
DATAEEEGEF437EEEA441EEEVA
DATAEEEGEFE438EEA441EEEVA
Cannot assign modification sites

Figure S2

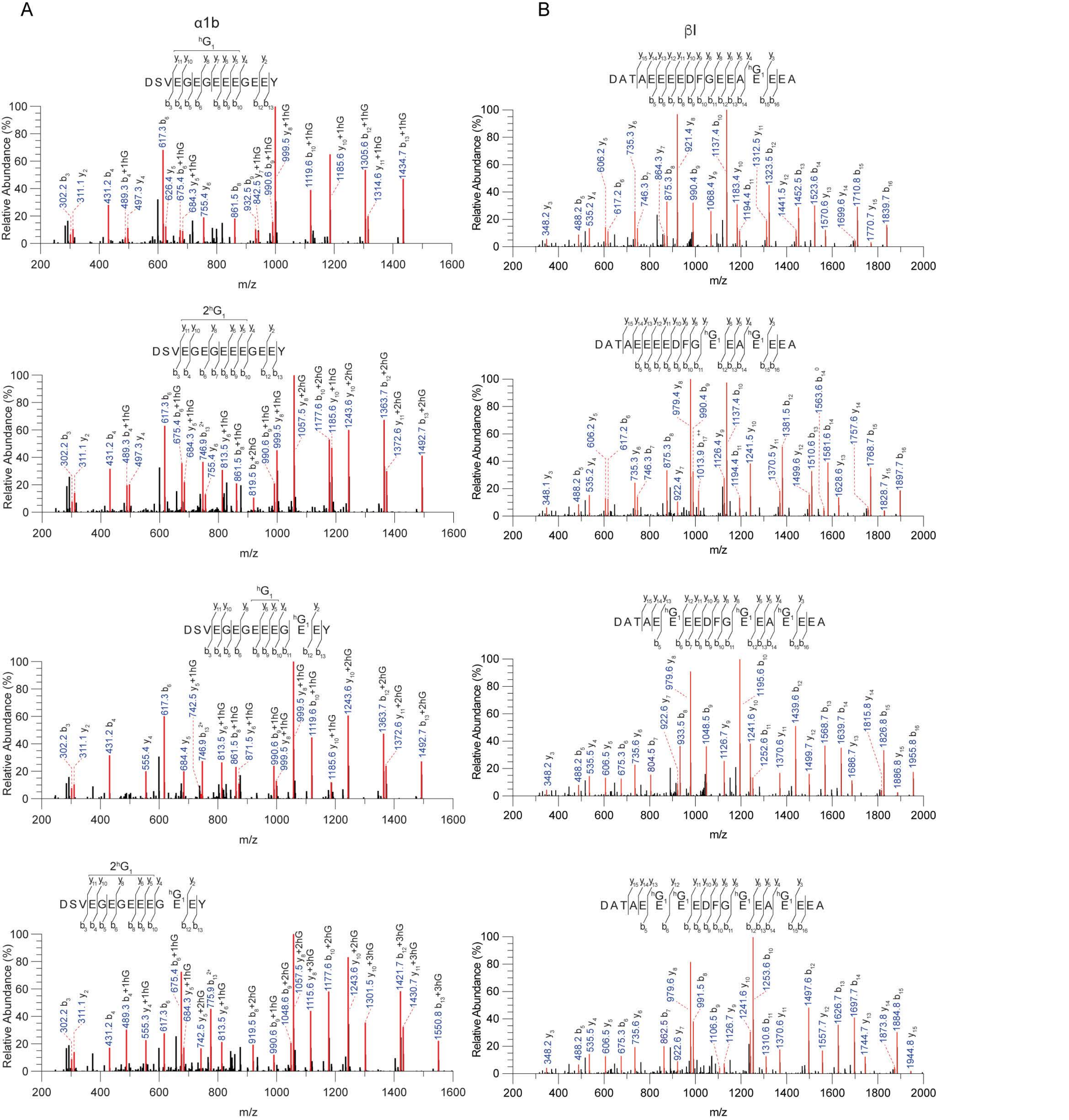
A
B
α1b
βI
DSVEGEGEEEGEEY
DATAEEEEDFGEEAEEEA
Relative Abundance (%)
m/z

Figure S3

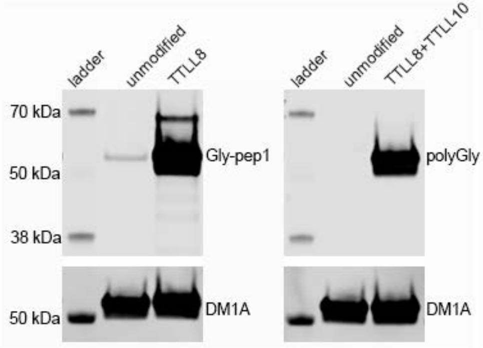
ladder
unmodified
TTLL8
ladder
unmodified
TTLL8+TTLL10
70 kDa
50 kDa
38 kDa
Gly-pep1
polyGly
50 kDa
DM1A
DM1A

Figure S4

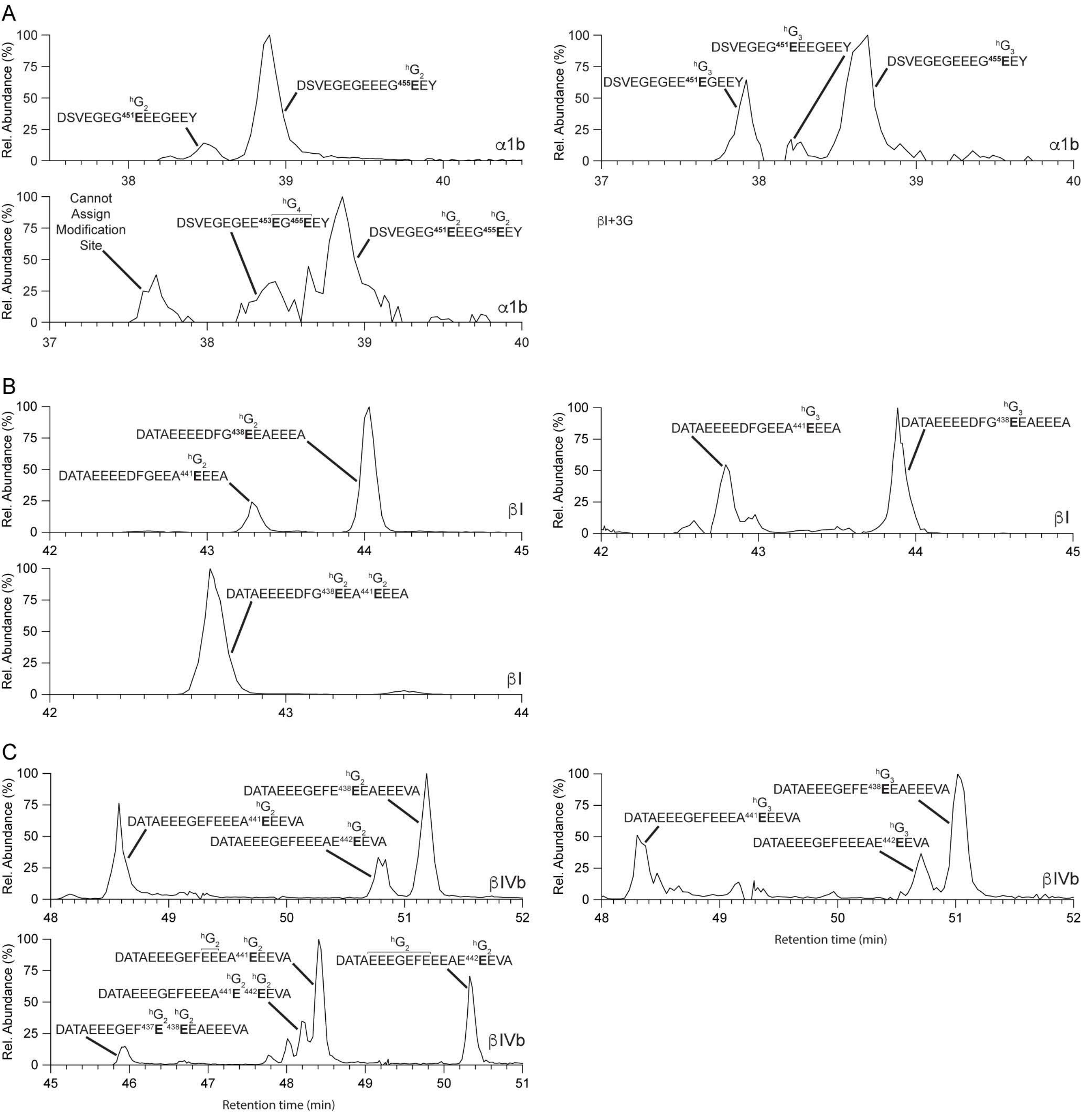
A
Rel. Abundance (%)
hG2
DSVEGEG451EEEGEEY
hG2
DSVEGEGEEEG455EEY
α1b
hG3
DSVEGEG451EEEGEEY
hG3
DSVEGEGEE451EGEEY
hG3
DSVEGEGEEEG455EEY
Cannot Assign Modification Site
hG4
DSVEGEGEE453EG455EEY
hG2 hG2
DSVEGEG451EEEG455EEY
βI+3G
B
hG2
DATAEEEEDFG438EEAEEEA
hG2
DATAEEEEDFGEEA441EEEA
βI
hG3
DATAEEEEDFGEEA441EEEA
hG3
DATAEEEEDFG438EEAEEEA
hG2 hG2
DATAEEEEDFG438EEA441EEEA
C
hG2
DATAEEEGEFE438EEAEEEVA
hG2
DATAEEEGEFEEEA441EEEVA
hG2
DATAEEEGEFEEEAE442EEVA
βIVb
hG3
DATAEEEGEFE438EEAEEEVA
hG3
DATAEEEGEFEEEA441EEEVA
hG3
DATAEEEGEFEEEAE442EEVA
hG2 hG2
DATAEEEGEFEEEA441EEEVA
hG2
DATAEEEGEFEEEAE442EEVA
hG2 hG2
DATAEEEGEFEEEA441E442EEVA
hG2 hG2
DATAEEEGEF437E438EEAEEEVA
Retention time (min)

bioRxiv preprint doi: https://doi.org/10.1101/2024.03.31.587457; this version posted August 4, 2025. The co
(which was not certified by peer review) is the author/funder. This article is a US Government work. It is not su
105 and is also made available for use under a CC0 license.

Figure S5

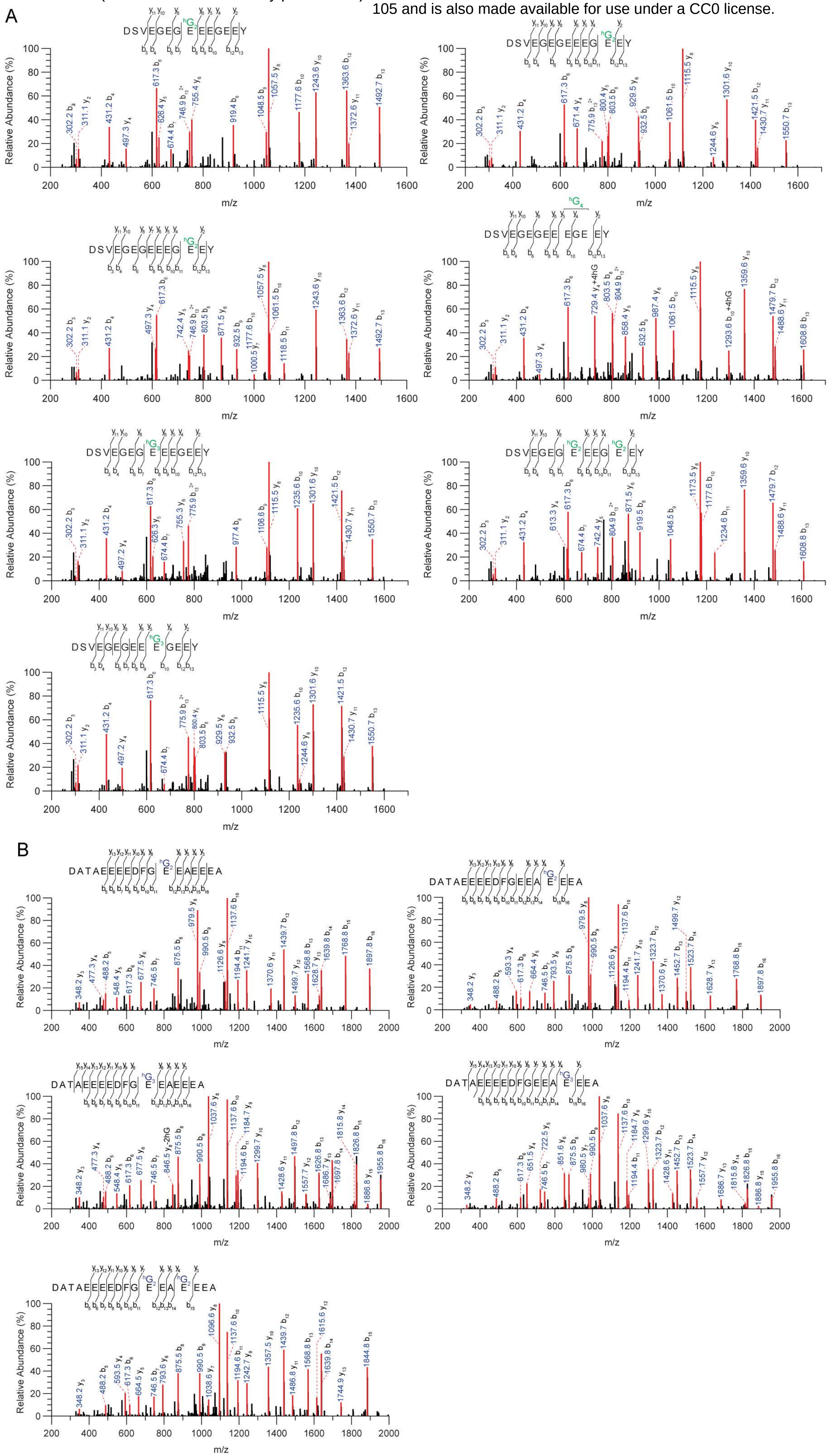

Figure S6

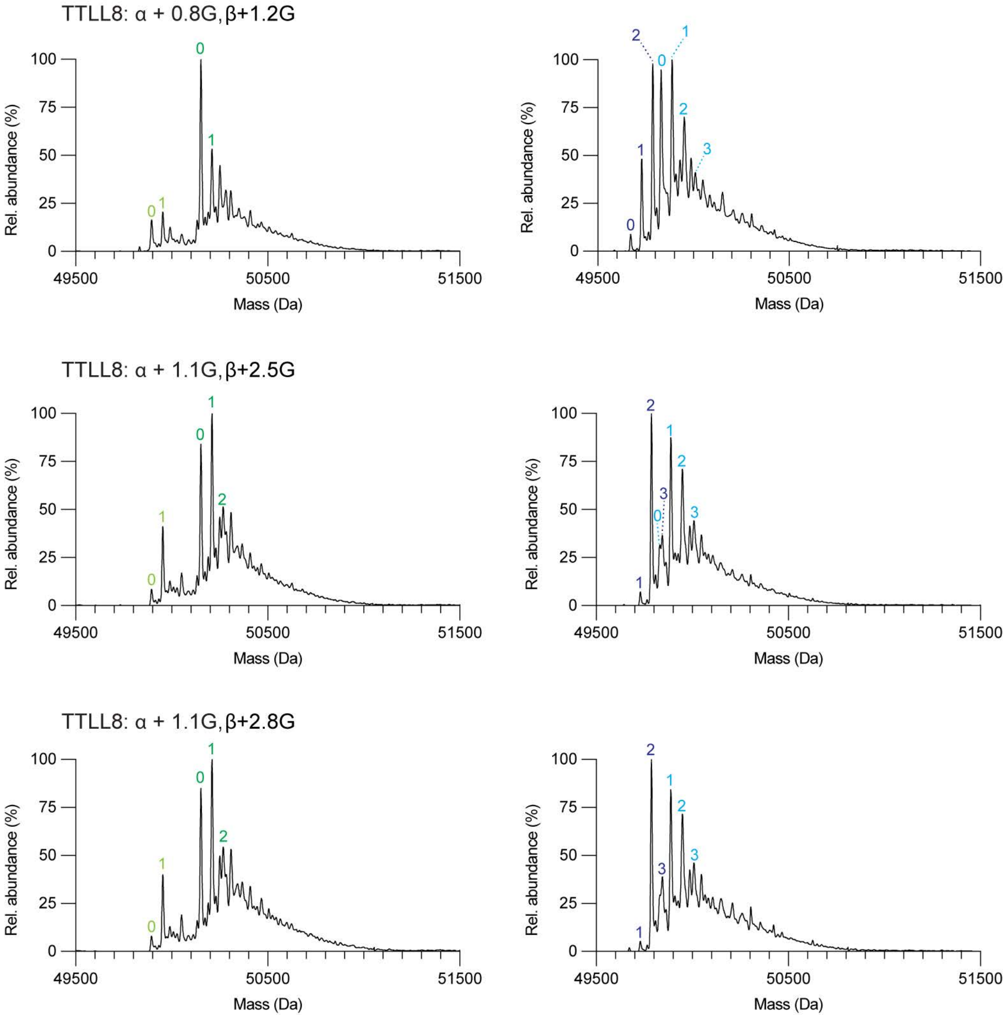

TTLL8: α + 0.8G, β+1.2G
TTLL8: α + 1.1G, β+2.5G
TTLL8: α + 1.1G, β+2.8G
Rel. abundance (%)
Mass (Da)
0
25
50
75
100
49500
50500
51500

Figure S7

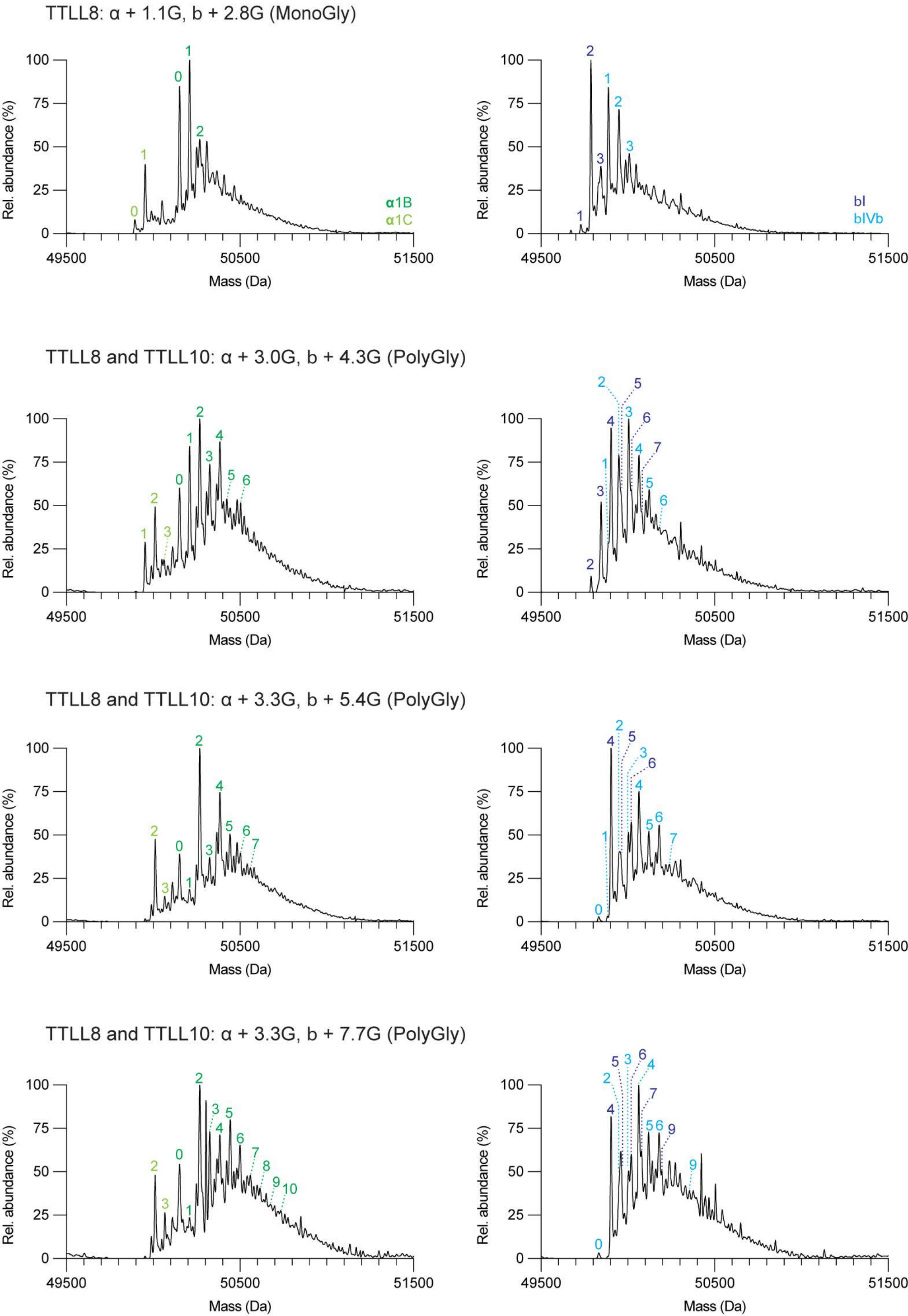
TTLL8: α + 1.1G, b + 2.8G (MonoGly)
TTLL8 and TTLL10: α + 3.0G, b + 4.3G (PolyGly)
TTLL8 and TTLL10: α + 3.3G, b + 5.4G (PolyGly)
TTLL8 and TTLL10: α + 3.3G, b + 7.7G (PolyGly)
α1B
α1C
bI
bIVb
Rel. abundance (%)
Mass (Da)
49500
50500
51500
0
25
50
75
100

Figure S8

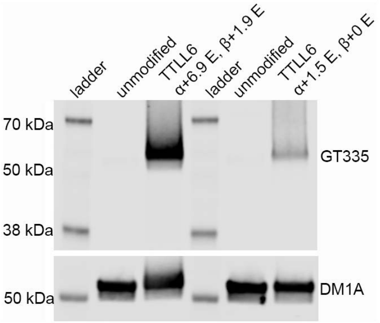
ladder
unmodified
TTLL6
α+6.9 E, β+1.9 E
ladder
unmodified
TTLL6
α+1.5 E, β+0 E
70 kDa
50 kDa
38 kDa
GT335
50 kDa
DM1A

Figure S9

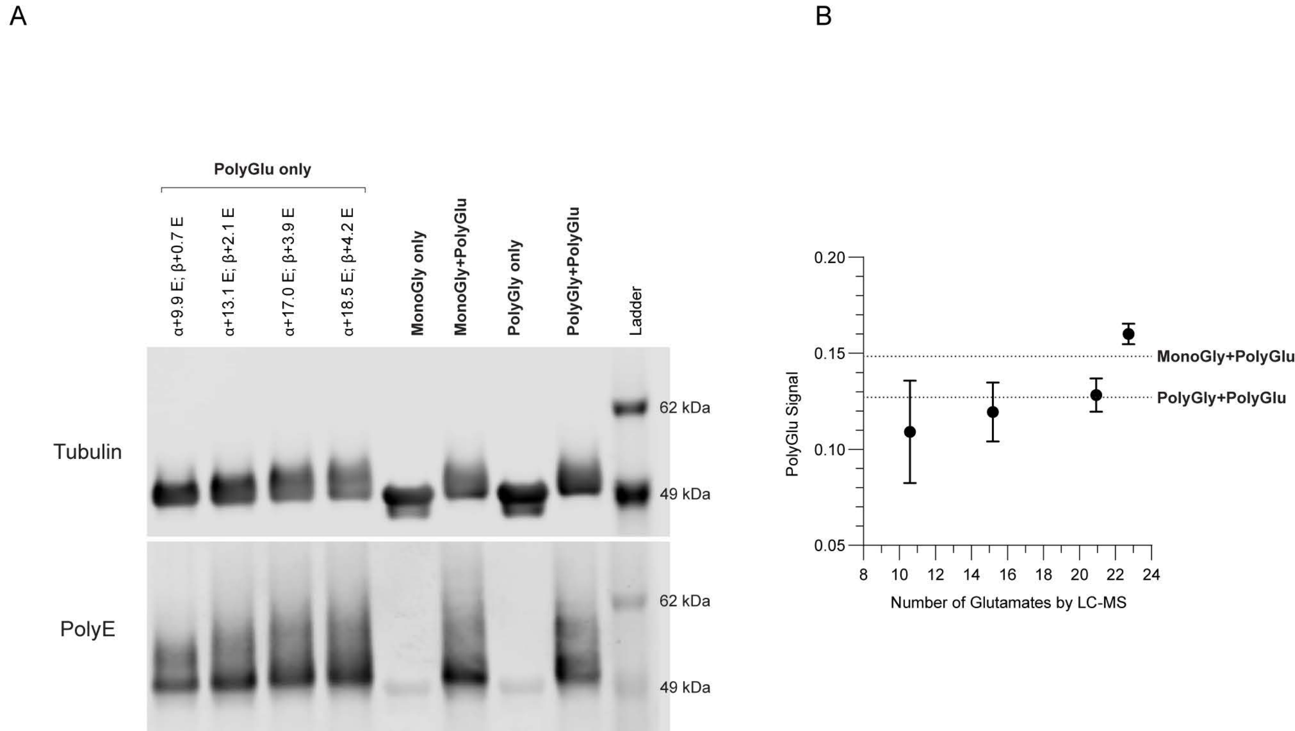
A
PolyGlu only
α+9.9 E; β+0.7 E
α+13.1 E; β+2.1 E
α+17.0 E; β+3.9 E
α+18.5 E; β+4.2 E
MonoGly only
MonoGly+PolyGlu
PolyGly only
PolyGly+PolyGlu
Ladder
Tubulin
PolyE
62 kDa
49 kDa
62 kDa
49 kDa
B
PolyGlu Signal
0.20
0.15
0.10
0.05
8
10
12
14
16
18
20
22
24
Number of Glutamates by LC-MS
MonoGly+PolyGlu
PolyGly+PolyGlu
